# Supplementary material for: Proteomic evidence for aerobic methane production in groundwater by methylotrophic Methylotenera
Source: ISME J. 2025 Feb 10;19(1):wraf024. doi: 10.1093/ismejo/wraf024 (PMC11978286; doi:10.1093/ismejo/wraf024)
Supplement: SI_wraf024 [file si_wraf024.docx]

**Supporting information for**

**Proteomic evidence for aerobic methane production in groundwater by methylotrophic *Methylotenera***

Shengjie Li^1,2^, Xiaoli Dong^1,3^, Pauline Humez^1^, Joanna Borecki^4^, Jean Birks^4^, Cynthia McClain^1,5^, Bernhard Mayer^1^, Marc Strous^1^, Muhe Diao^1,6^*

^1^Department of Earth, Energy and Environment, University of Calgary, Calgary, AB T2N 1N4, Canada

^2^Max Planck Institute for Marine Microbiology, Bremen 28359, Germany

^3^Provincial Laboratory for Public Health, Calgary, AB T2N 4W4, Canada

^4^Alberta Environment and Protected Areas, Calgary, AB T2L 2K8, Canada

^5^Alberta Biodiversity Monitoring Institute, University of Alberta, Edmonton, AB T6G 2E9, Canada

^6^State Key Laboratory of Pollution Control and Resources Reuse, College of Environmental Science and Engineering, Tongji University, Shanghai 200092, P.R. China

*corresponding author

Email address: [mdiao@tongji.edu.cn](mailto:mdiao@tongji.edu.cn); [muhe.diao@ucalgary.ca](mailto:muhe.diao@ucalgary.ca)


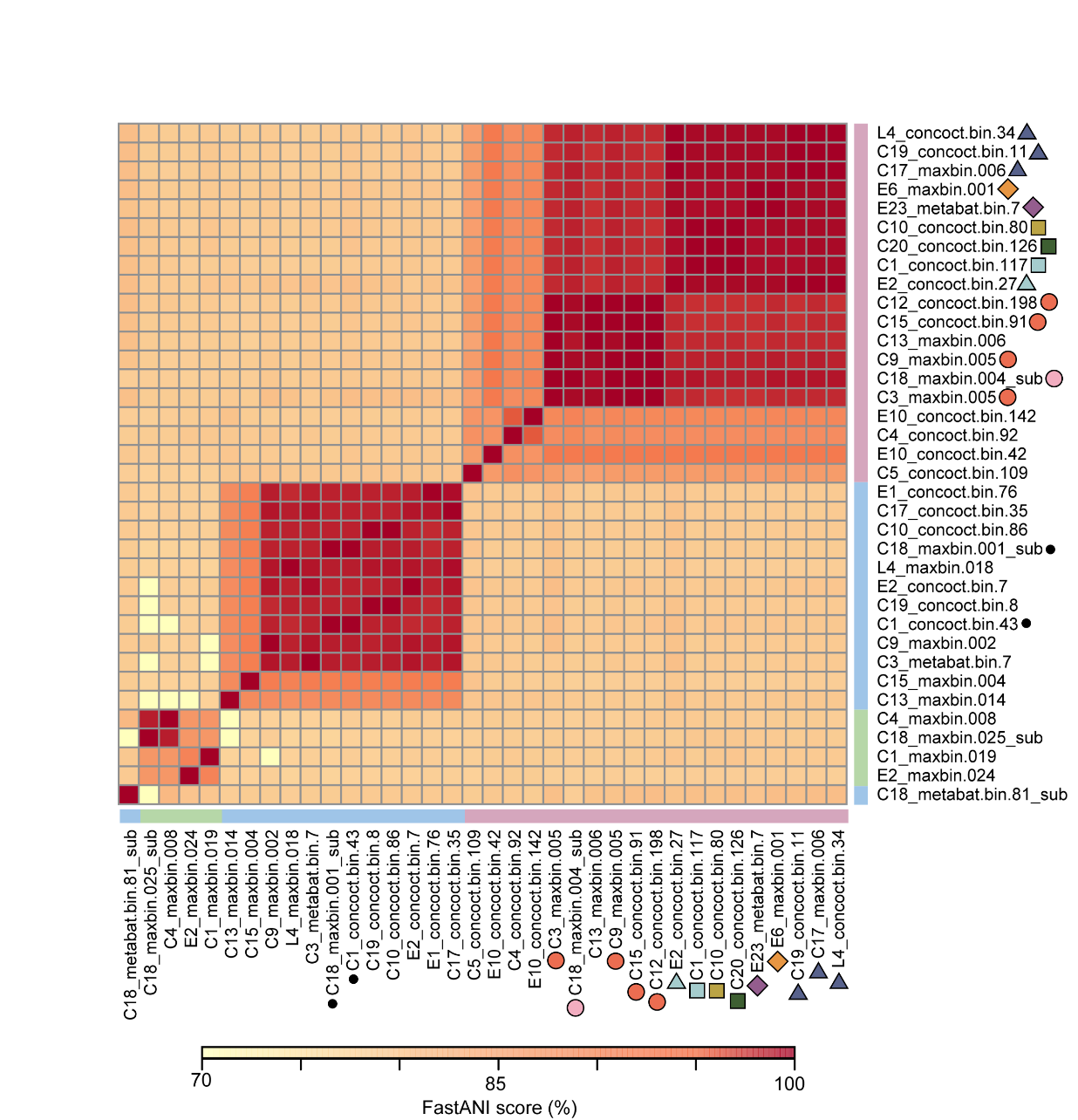


Figure S1. **Heatmap depicting average nucleotide identity (ANI) between *Methylotenera* metagenome-assembled-genomes (MAGs) calculated with FastANI**. Each cell represents a pairwise comparison between the named MAGs on the rows and columns. Markers correspond to the five populations consisting of MAGs with >99.9% ANI (Figure 1b). Color bars correspond to the three clades of groundwater *Methylotenera* (pink G1, green G2, blue G3, Figure 2a).


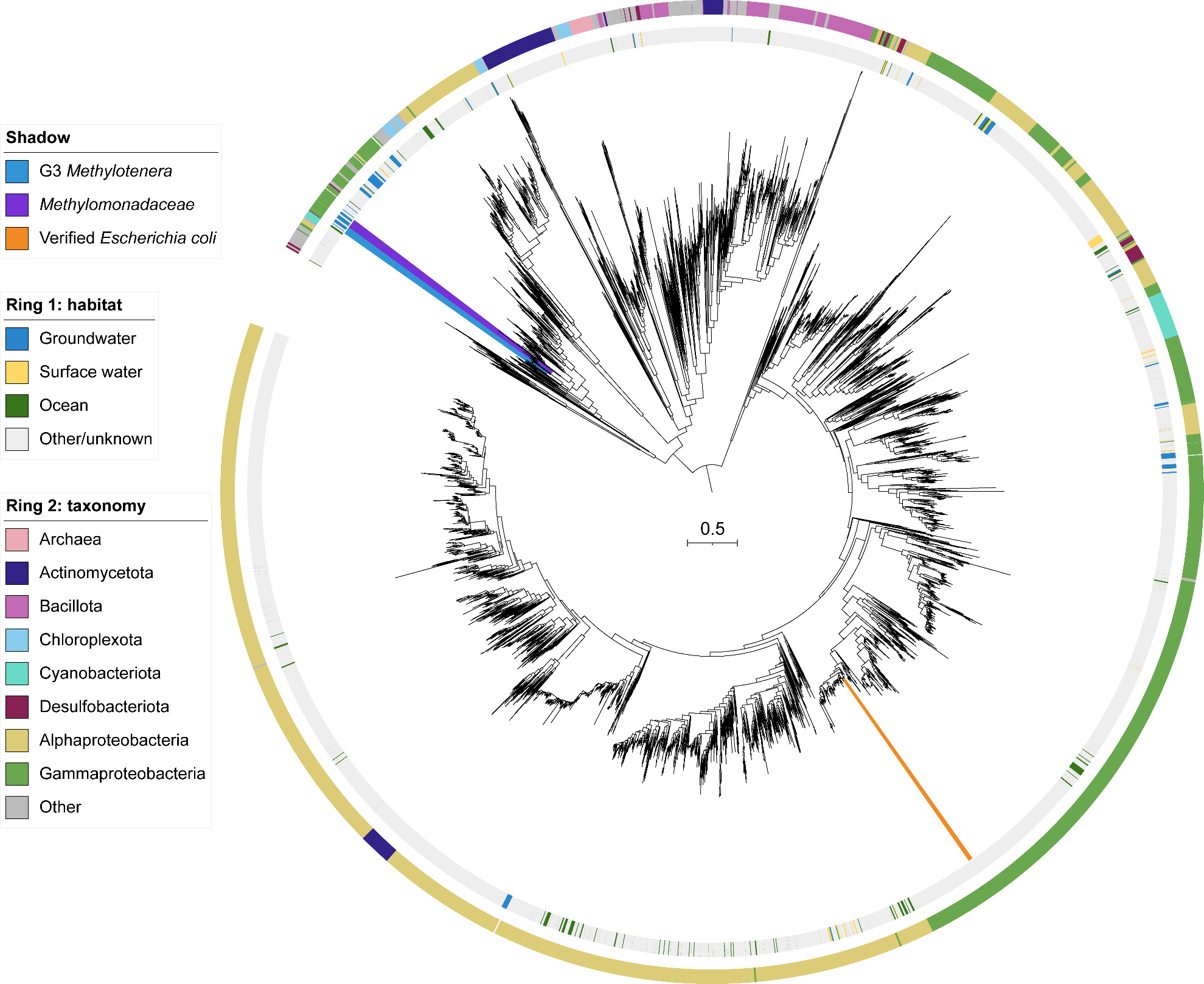


Figure S2. **Phylogenetic tree of the C-P lyase core complex subunit J (PhnJ)**. PhnJ sequences retrieved from this study and the reference database are included (Supplementary Table S7). Blue and purple shadows on leaves indicate those from G3 *Methylotenera* and *Methylomonadaceae* which are mostly retrieved from this study. Gold shadow indicates those experimentally verified PhnJ sequences in *Escherichia* *coli*. From inside to outside, the two rings around the tree indicate: (1) habitat and (2) taxonomy of the genome.


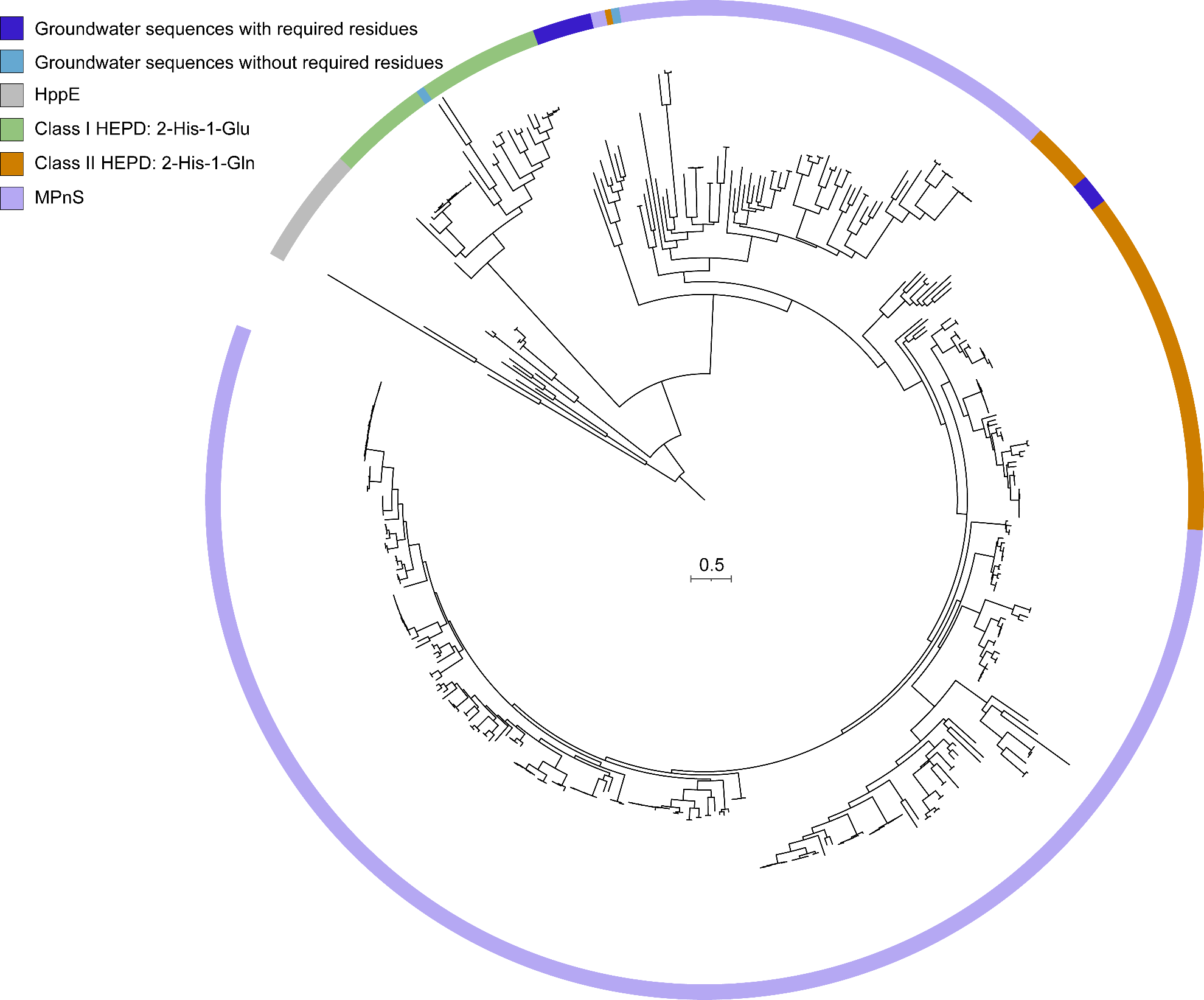


Figure S3. **Phylogenetic tree of the methylphosphonate synthase (MPnS)**. MPnS like sequences retrieved from this study and the reference sequences obtained from a previous study (Born et al., 2017) are included (Supplementary Table S8). The presence of the 2-histidine-1-glutamine iron-coordinating triad and the two glutamine-adjacent residues (phenylalanine and isoleucine) required for methylphosphonate synthesis (Born et al., 2017) were checked (Supplementary dataset 1). The ring around the tree indicates the groundwater sequences and the annotated reference sequences.
